# Overcoming Antibiotic Resistance and Treating Bacterial Infections with Biological Nanoparticles

**DOI:** 10.3390/ijms262411780

**Published:** 2025-12-05

**Authors:** Boris Ponomarev, Natalia Ponomareva, Artyom Kachanov, Konstantin Evmenov, Sergey Brezgin, Anastasiia Kostyusheva, Vladimir Chulanov, Peter Timashev, Dmitry Kostyushev, Alexander Lukashev

**Affiliations:** 1 Laboratory of Genetic Technologies, Martsinovsky Institute of Medical Parasitology, Tropical and Vector-Borne Diseases, Sechenov University, Moscow 119435, Russia; botiska5@gmail.com (B.P.); ponomareva.n.i13@yandex.ru (N.P.); kachanov.av99@gmail.com (A.K.); kevmenov@mail.ru (K.E.); seegez@mail.ru (S.B.); kostyusheva_ap@mail.ru (A.K.); 2 Engelhardt Institute of Molecular Biology, Russian Academy of Science, Moscow 119991, Russia; vladimir@chulanov.ru; 3 Laboratory of Experimental Therapy of Infectious Diseases, Martsinovsky Institute of Medical Parasitology, Tropical and Vector-Borne Diseases, Sechenov University, Moscow 119435, Russia; 4 Department of Infectious Diseases, Sechenov University, Moscow 119435, Russia; 5 Institute for Regenerative Medicine, I.M. Sechenov First Moscow State Medical University, Moscow 119991, Russia; timashev_p_s@staff.sechenov.ru; 6 Faculty of Bioengineering and Bioinformatics, Lomonosov Moscow State University, Moscow 119192, Russia; 7 Department of Biotechnology, Sechenov University, Moscow 119415, Russia; 8 Martsinovsky Institute of Medical Parasitology, Tropical and Vector-Borne Diseases, Sechenov University, Moscow 119435, Russia; alexander_lukashev@hotmail.com; 9 Research Institute for Systems Biology and Medicine, Moscow 117246, Russia

**Keywords:** antibiotic resistance, drug delivery, exosomes, extracellular vesicles, outer membrane vesicles

## Abstract

For over eight decades, antibiotics have been the cornerstone of treating bacterial infections. However, the rapid rise of antibiotic-resistant pathogens has created an urgent need for alternative therapeutic strategies. Advances in nanotechnology offer a promising solution through the development of bio-derived nanoparticles. This broad class includes extracellular vesicles such as exosomes and bacterial outer membrane vesicles (OMVs), as well as bioengineered cell membrane-coated nanoparticles (CMNPs) that combine synthetic cores with natural membranes from diverse source cells. These particles possess unique physicochemical and biological properties, such as intrinsic bioactivity, biocompatibility, and structural versatility, that can be harnessed for antimicrobial therapy. This review synthesizes recent progress in the design, characterization, and application of biological nanoparticles for combating bacterial infections. We place particular emphasis on their mechanisms of action, therapeutic potential, and key research directions that could accelerate their translation into clinical use.

## 1. Introduction

Throughout human history, microorganisms have been both constant companions and formidable adversaries. The mid-20th-century discovery of antibiotics offered a powerful solution, effectively controlling bacterial infections and saving countless lives. However, the global overuse of these drugs has driven the rapid selection and spread of antibiotic-resistant mutations [1]. This escalating crisis presents a critical challenge: overcoming resistance requires either the costly and labor-intensive modification of existing antibiotics or the discovery of novel classes, both demanding consolidated efforts across the global research community [2]. Consequently, the risk of rising mortality from previously treatable infections is increasing. In 2019, bacterial antimicrobial resistance (AMR) was directly responsible for an estimated 1.27 million deaths and contributed to nearly 4.95 million total deaths [2].

The limitations of conventional antimicrobial agents themselves present a formidable clinical challenge. Administered at standard therapeutic doses, even essential antibiotics can provoke significant adverse effects, including drug fever, cutaneous eruptions, anaphylaxis, and glomerular toxicity, which directly compromise patient safety and treatment tolerability [3]. Furthermore, pathogenic bacteria often form biofilms—complex, extracellular polymeric matrices that can elevate resistance up to 1000-fold compared to their free-floating counterparts. Functioning as physical barriers, biofilms impede antimicrobial penetration and serve as hubs for horizontal gene transfer, thereby accelerating the spread of resistance [3]. In light of these issues, classical antimicrobial drugs are increasingly insufficient, particularly against multidrug-resistant infections.

In response, nanomedicine has emerged as a promising frontier. Nanoparticle (NP)-based delivery systems are being extensively investigated to enhance therapeutic efficacy by protecting drugs from degradation, promoting their targeted accumulation at infection sites, and reducing off-target toxicity [4].

Synthetic NPs, such as polymeric nanoparticles, liposomes, dendrimers, metallic nanostructures (e.g., gold or silver NPs), and inorganic platforms like mesoporous silica, offer tunable size, charge, and surface chemistry, but they face challenges related to rapid opsonization, immune clearance, and limited intrinsic targeting. By contrast, a particularly promising class of NPs is derived from biological sources.

Beyond the general advantages of nanoscale drug-carrier systems, biologically derived NPs demonstrate unique structural and functional attributes that directly address the limitations inherent to synthetic nanomaterials. The utilization of NPs derived from natural cell membranes, such as exosomes, exosome-mimetic nanovesicles, hybrid nanovesicles, and cell membrane-coated nanostructures, represents a cutting-edge avenue for antimicrobial therapy [5,6,7,8,9,10,11]. These biological NPs inherit native membrane architectures, which enable targeting capabilities and high biocompatibility, making them ideal platforms for developing sophisticated, targeted antimicrobial strategies (Table 1).

A promising technology centered on biomimetic NPs, synthetic NPs enveloped in biological or synthetic membranes, is gaining significant traction across various medical fields [25]. These cell membrane-coated NPs constitute a versatile platform in nanomedicine, boasting unique properties that enable precise, targeted therapy and a marked ability to evade the innate immune system, which often neutralizes conventional nanocarriers [26,27]. By preserving intrinsic membrane proteins and receptors, their biomimetic design allows them to avoid immune clearance, neutralize bacterial toxins, and directly target pathogenic bacteria. In the context of antibacterial therapy, this approach leverages diverse biological vesicles. These include exosomes, which are single-membraned vesicles secreted by host cells and enriched with proteins, lipids, and nucleic acids, and outer membrane vesicles (OMVs), which are bilayered nanostructures naturally released by Gram-negative bacteria. The application of these biological NPs has already been demonstrated in the development of innovative vaccines and targeted delivery systems.

This review systematically outlines the primary classes of biological NPs and explores their potential applications in treating bacterial infections.

## 2. Mechanisms of Antibiotic Resistance

**Figure 1:**
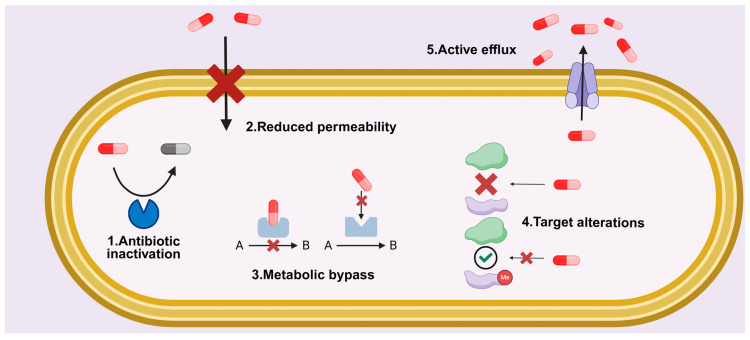
Principal molecular mechanisms of bacterial antibiotic resistance. The figure depicts five key strategies: (1) Antibiotic inactivation: enzymatic modification or degradation of the antibiotic molecule. (2) Reduced permeability: decreased antibiotic penetration into the cell via modification of porins or other channels. (3) Metabolic bypass: utilization of alternative metabolic pathways unaffected by the antibiotic. (4) Target modification: structural alteration of the antibiotic’s cellular target, preventing drug binding. (5) Active efflux: energy-dependent extrusion of the antibiotic from the cell via efflux pumps. Created in BioRender. Kachanov, A. (2025) https://BioRender.com/fc8qt63.

Bacteria possess a remarkable capacity to evolve resistance to antimicrobial agents through genetic mutations over time [28]. There are several principal mechanisms that interfere with the classical treatment of bacterial infections (Figure 1). Certain bacteria synthesize enzymes that chemically modify or degrade antimicrobial compounds, rendering them inactive. For example, β-lactam antibiotics (e.g., penicillin) are inactivated by bacterial β-lactamases through the cleavage of the β-lactam ring [29]. Acetyl transferase, phosphotransferase, and adenyl transferase enzymes are other examples of enzymes that can render certain antibiotics inactive [12,30,31]. Mutations or post-translational modifications in antibiotic-binding sites of bacterial targets can reduce drug affinity without impairing essential cellular functions. In the case of resistance to erythromycin, for example, methylation of the adenine residue in the peptidyl transferase of 23S ribosomal RNA reduces the enzyme’s affinity to the antibiotic without disrupting protein synthesis. The modification of penicillin-binding proteins is another example of this resistance mechanism. These proteins catalyze the formation of cross-links in peptidoglycan, a crucial component of the bacterial cell wall that provides structural support, but bacteria can acquire modified versions of these proteins, thus reducing the binding affinity of β-lactam antibiotics [15]. Certain structural changes in the cell surface membranes can reduce their permeability to antibiotics. For example, a reduction in the number of porins or a mutation in the porin gene can decrease permeability and cause resistance to antibiotics in Gram-negative bacteria [14]. In addition, the energy-driven drug efflux systems provide the expulsion of the drug outside the cell. These systems eliminate antibiotics that have been absorbed by bacterial cells, lowering intracellular drug concentrations. Many resistant strains overexpress such molecular transporters, increasing their capacity for drug expulsion [13]. In the pursuit of antibiotic resistance, certain bacterial strains have developed the ability to activate alternative metabolic routes when a primary pathway is blocked. For example, bacteria exposed to sulfonamides are still able to produce folic acid via an alternative enzymatic pathway [32]. Furthermore, bacteria have been observed to establish systems of intercellular communication that facilitate the transfer of resistance genes. Consequently, this results in rapid dissemination of resistance traits within bacterial communities [33]. Moreover, the formation of extracellular polymeric substances (biofilms) is a phenomenon that enhances antibiotic resistance. Biofilms can impede drug penetration, promote horizontal gene transfer, and electrostatic repulsion within biofilm structures can hinder the diffusion of certain antibiotics, such as aminoglycosides [3].

## 3. Biological NPs

Considering the numerous strategies utilized by bacteria to circumvent or neutralize antibiotics, it is essential to develop therapeutic platforms capable of bypassing or directly neutralizing these resistance mechanisms. One emerging approach involves the exploitation of extracellular vesicles (EVs), a heterogeneous class of nanoscale structures naturally secreted by cells, including exosomes, outer-membrane vesicles, and cell membrane-coated NPs [34].

Exosomes are a subset of EVs composed of a membrane layer and having a diameter of 30–150 nm. They form via inward budding of the endosomal membrane, produced from multivesicular bodies (MVBs), so-called intraluminal nanovesicles. MVBs then fuse with the plasma membrane to release exosomes into extracellular space. Exosomes are secreted by a broad range of eukaryotic cells and display a lipid bilayer enriched with tetraspanins (CD9, CD63, CD81), adhesion molecules, and cargo molecules, including proteins, lipids, metabolites, and nucleic acids. Their endogenous origin confers low immunogenicity and allows ligand–receptor specificity for targeted intercellular communication [34,35]. However, the purification and standardization of both the size and quality parameters of these particles, despite significant advances in large-scale manufacturing and quality control, remain challenging at present [36].

**Figure 2:**
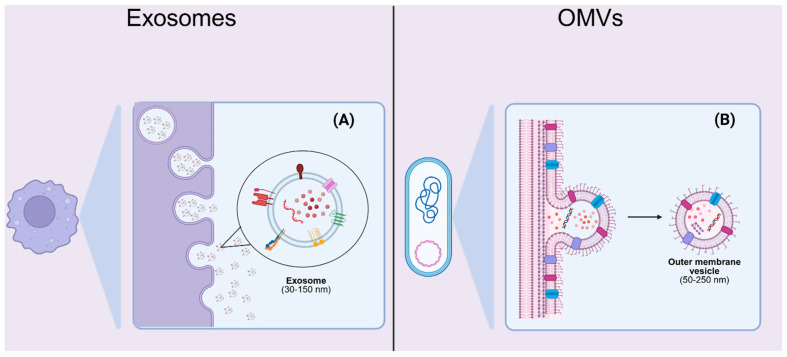
Schematic representation of exosomes and outer membrane vesicles (OMVs). (**A**) Exosomes are nanosized extracellular vesicles (30–150 nm) secreted by eukaryotic cells through the endosomal pathway. Exosomes carry a cargo of nucleic acids (mRNA, miRNA, and other noncoding RNAs), proteins (including adhesion molecules and signaling mediators), and lipids. They also display characteristic surface markers such as the tetraspanins CD9, CD63, and CD81. Their inherent ability to transfer functional biomolecules has been exploited for drug delivery, vaccine development, and nucleic acid transport. (**B**) OMVs are nanoscale vesicles (50–250 nm) secreted by Gram-negative bacteria via outer membrane blebbing. They encapsulate components of the bacterial envelope, including lipopolysaccharides (LPS), outer membrane proteins, periplasmic enzymes, and fragments of DNA or RNA. OMVs have been studied for applications in antibacterial drug delivery, vaccine design, and immunomodulation. Together, exosomes and OMVs represent biologically derived vesicles with distinct origins and molecular compositions that can be harnessed for therapeutic purposes. Created in BioRender. Kachanov, A. (2025) https://BioRender.com/eyoi5on.

OMVs are spherical bilayered vesicles (50–250 nm) shed by Gram-negative bacteria via blebbing of the outer membrane. They encapsulate periplasmic components and contain lipopolysaccharides (LPS), outer membrane proteins (OMPs), phospholipids, peptidoglycan fragments, and bacterial DNA/RNA (Figure 2). While OMVs can elicit robust immunological responses and serve as vehicles for antibiotic delivery, their endotoxin-associated immunotoxicity requires careful engineering to mitigate adverse effects in host tissues. Thus, further research is required to address the immunotoxicity of these substances to the patient and the challenges associated with their controlled manufacturing [37].

**Figure 3:**
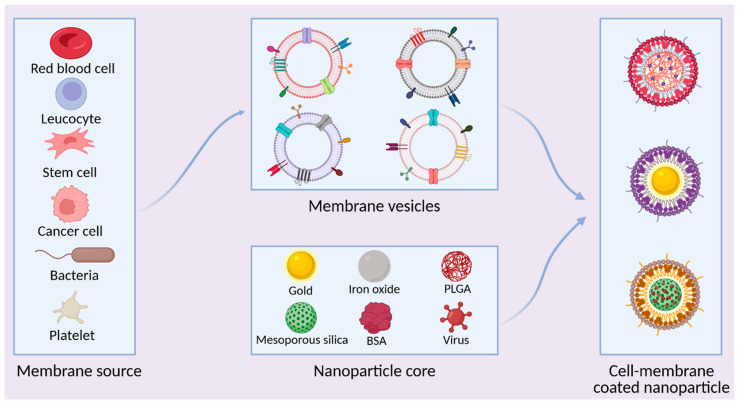
Schematic depicting of fabrication and characteristics of cell membrane-coated nanoparticles (CMNPs). Biological membranes derived from diverse sources—including red blood cells, leukocytes, stem cells, cancer cells, bacteria, platelets, and viruses—are isolated and used to coat synthetic nanoparticle cores (such as gold, iron oxide, mesoporous silica, poly(lactic-co-glycolic acid) (PLGA), or Bovine serum albumin (BSA)). The resulting CMNPs inherit the surface proteins and biological functionalities of their parental membranes, while maintaining the physicochemical properties of the nanoparticle core, thereby enabling immune evasion, targeted delivery, and multifunctional antibacterial activity. Created in BioRender. Kachanov, A. (2025) https://BioRender.com/8fe1sjk.

The last category of NPs discussed in this article pertains to cell membrane-coated NPs. These hybrid entities represent synthetic–biological hybrid systems, in which polymeric, metallic, or inorganic NPs cores are cloaked with plasma membranes from specific cell types, including erythrocytes, leukocytes, platelets, bacterial cells, or even tumor cells [21]. This biomimetic camouflage preserves surface protein orientation, receptor–ligand binding capabilities, and major histocompatibility complex (MHC) or pathogen-associated molecular pattern (PAMP) profiles (Figure 3). Such properties enable cell membrane-coated NPs to evade opsonization, thus prolonging systemic circulation, and deliver therapeutic cargo with pathogen- or tissue-specific tropism [27]. This class of NPs stands out for its high degree of modular design, allowing for independent optimization of the core material, the source of the surface membrane, and the presentation of functional ligands. This tunability enables the precise engineering of particles with tailored pharmacokinetics, specific targeting capabilities, and multimodal therapeutic functions. A significant barrier to the clinical translation of this promising platform, however, lies in its limited scalability, which is impeded by inefficient membrane extraction, low vesicle-core fusion yields, and a lack of scalable, economical manufacturing processes.

## 4. Exosomes

### 4.1. Antibiotic Delivery Based on Exosomes

Exosomes in natural environments are employed by cells for the delivery of diverse molecules and for facilitating cell-to-cell communication. The molecules delivered via this intrinsic transport can vary and include proteins, lipids, and metabolites, as well as a set of nucleic acids consisting of microRNAs, tRNA fragments, mRNAs, small RNA transcripts and RNA–protein complexes, mitochondrial DNA, and chromosomal DNA. Recent studies have demonstrated the efficacy of exosome-based antibiotic molecule delivery systems. For example, murine macrophage-derived exosomes were synthesized and subsequently loaded with the free oxazolidinone antibiotic linezolid by coincubation at 37 °C for 1 hour (the loading capacity of the exosomes was 5.06 ± 0.45%). Compared with the free drug, antibiotic-loaded exosomes were more efficiently internalized by Methicillin-resistant *Staphylococcus aureus*-infected macrophages, producing superior bacterial clearance both in vitro and in vivo with negligible cytotoxicity [38].

Furthermore, it has been posited that the administration of exosomes, loaded with the antibiotic Rifampicin (RIF), may offer advantages in tuberculosis infection of the central nervous system. The exosomes were derived from mesenchymal bone marrow stem cells, loaded with Rifampicin with the encapsulation efficiency of 18.8 ± 2.4%, and further modified with a targeting peptide Angiopep-2 (ANG), which enhanced their binding affinity to the blood–brain barrier. This facilitated an elevated concentration of Rifampicin in the brain, thereby potentiating the therapeutic efficacy against *Mycobacterium tuberculosis*. The study demonstrated the efficient passage of the blood–brain barrier in vitro model. The transport ratio of the ANG-Exo-RIF was 52.11 ± 5.68%, which was two-fold higher than the nontargeted Exo-RIF (23.82 ± 3.18%) and four-fold higher than the free RIF (11.4 ± 1.25%). Furthermore, the ANG-Exo-Rif exosomal complex demonstrated effective antibacterial properties, albeit exclusively in vitro [39].

### 4.2. Exosome-Based Antibacterial Vaccines

In addition to drug delivery, it is possible to utilize exosomes as antigen carriers to facilitate the development of immunity to various bacterial infections, whether as prophylaxis or as a therapeutic measure. For instance, it has been demonstrated that *Mycobacterium tuberculosis* var. *bovis* BCG can be utilized to synthesize exosome vaccines against the infection caused by *Mycobacterium tuberculosis*. Macrophages sensitized by *M. bovis* BCG released exosomes, which carried mycobacterial antigens on the membrane surface and were able to stimulate T-cell response. Moreover, the exosome exhibited the presence of MHC class I and II proteins, as well as costimulatory molecules on its surface, thereby facilitating efficient activation and stimulation of CD4+ helper T and CD8+ cytotoxic T-cell maturation. Following the administration of exosomes carrying BCG antigens, the isolated immune cells demonstrated the capacity to synthesize interferon-gamma (IFN-γ), thereby indicating the efficacy of the immunization process [40].

## 5. OMVs

### 5.1. Application of OMVs as a Delivery Instrument

Gram-negative bacteria are distinguished by their unique cell wall structure, comprising an inner membrane, a thin peptidoglycan layer, and an outer membrane enriched with LPS. The outer membrane can produce a specific type of EVs with a diameter ranging from 50 to 250 nm that are known as OMVs and play a crucial role in various biological processes. They encapsulate periplasmic constituents and recapitulate the antigenic and biochemical profile of their parent bacterium, including outer membrane proteins (OMPs), phospholipids, lipooligosaccharides, peptidoglycan fragments, nucleic acids, and virulence factors [41,42,43]. Among other applications, these vesicles have been repurposed for antibiotic delivery systems and subunit vaccine platforms [37].

OMVs have been identified as a promising platform for the delivery of antibacterial molecules to a specific target, with specificity determined by surface-expressed antigens and adhesins. For instance, vesicles with antibiotic Levofloxacin were obtained from an *Acinetobacter baumannii* culture through the utilization of the bacterial efflux pump system. The LEV-OMVs were prepared by incubating the bacteria at 1/8 of the minimum inhibitory concentration (MIC) and contained levofloxacin at a concentration that was 18.43-fold greater than that in the medium due to the function of bacterial efflux pumps. Analysis of the Lev-OMV NPs demonstrated satisfactory biocompatibility and broad-spectrum bactericidal efficacy against *Pseudomonas aeruginosa*, *Klebsiella pneumoniae* and *Escherichia coli* bacteria in vivo. Furthermore, the NPs exhibited site-retention properties that limited systemic drug dispersion and reduced off-target toxicity [44].

Additionally, it was feasible to generate hybrid OMVs by coating mesoporous silica NPs pre-loaded with rifampicin using *E. coli*-derived OMVs. The system retained the antigenic identity of their bacterial source, thereby conferring homotypic targeting, and demonstrated biocompatibility in both in vivo and in vitro settings [24].

An additional feature of OMVs that is potentially usable in combating infections is their inherent antibacterial properties, e.g., due to murein hydrolases that can interfere with the function of the cell wall of target bacteria. However, this direction is still at a very early stage.

Therefore, OMVs can be used to efficiently deliver antibiotics. However, being composed of a bacterial cell wall, they are inherently rich in PAMPs and toxic molecules, difficult to produce and standardize at an industrial scale; therefore, their clinical use remains questionable.

### 5.2. OMV as a Vaccination Instrument

The antigenic surface composition of OMVs, notably LPS O-antigen chains, Outer Membrane Proteins (OMPs) and strain-specific virulence determinants enable their use as non-replicating immunogens. Because OMVs lack replicative capacity, the risk of infection is minimized, while their PAMPs strongly engage innate immune receptors such as TLR2, TLR4, and NOD-like receptors. Accordingly, a recent study has demonstrated the efficacy of control measures for *Pseudomonas aeruginosa*, the bacterium responsible for opportunistic, life-threatening infections. For this purpose, OMVs with *Pseudomonas aeruginosa* antigens embedded in the membrane–PcrV-HitAT complexes–were created based on a recombinant strain of *Yersinia pseudotuberculosis*. Immunization induced a balanced immune response, specifically in Th1/Th2 cells, and prevented 73% of lethal bacterial infections [16]. Furthermore, the efficacy of intranasal immunization with OMVs derived from *Acinetobacter baumannii* resulted in the development of immunity in mice and complete recovery after bacterial challenge [17].

Moreover, OMV NPs based on PEGylated nano-*Rehmannia glutinosa* polysaccharide functioned as an effective adjuvant. Dendritic cells were shown to successfully ingest these NPs, which resulted in the enhancement of the proliferation of both CD8+ and CD4+ T cells. Furthermore, specific antibodies were identified in the group administered with OMVs, providing the most direct evidence that NPs can effectively induce a prolonged bacteria-specific antibody response [18].

Recent studies have also demonstrated the use of hybrid systems for immunization. For instance, a multiantigenic nanovaccine against infection caused by *Pseudomonas aeruginosa* was developed. The formation of hybrid NPs was initiated by the assembly of gold NPs, which were then encircled by membranes comprising OMV elements and vesicles derived from macrophage membranes. In addition, secreted *P. aeruginosa* peptides were incorporated into this milieu by coincubation. Consequently, NPs that possessed bacterial antigenic surfaces and secreted toxins demonstrated high prophylactic efficacy against *P. aeruginosa*-induced septicemia, resulting in a 100% survival rate in mice. Hybridisation of OMVs and macrophage membranes further reduced inflammation and cellular toxicity in both in vivo and in vitro models [19].

### 5.3. Additional Therapeutic Applications of OMVs

Emerging applications for OMVs extend beyond direct antimicrobial delivery and immunization. For instance, one innovative concept involves a bacteriostatic implant fabricated from *E. coli*-derived OMVs and a poly(tannic acid) coating. This implant selectively inhibited the growth of heterologous bacterial strains while permitting the growth of homologous bacteria, and significantly enhanced wound healing in vivo. The underlying mechanism appears to involve the upregulation of antioxidative stress genes in the inhibited (heterologous) bacteria and the activation of biofilm-related genes in the non-targeted (parental) strains, explaining the observed selective antimicrobial effect [20].

## 6. Cell Membrane-Coated NPs and Their Types

Advances in nanotechnology have enabled the fabrication of NPs from the biological membranes of a diverse range of cells [45]. These biomimetic NPs combine the tunable physicochemical properties of a synthetic core with the complex biological functions of their source cells. By preserving the native structure of membrane components, including transmembrane proteins, glycosylation patterns, and lipid asymmetry, this cloaking technique facilitates critical bio-interactions such as immune evasion, targeted adhesion, and traversal of biological barriers [19]. The classification of cell membrane-coated NPs is principally defined by the origin of their source membrane:

The first type is derived from red blood cells (RBCs). The specific pattern of RBC membranes, represented by CD47+, sialic acid, peptides, and glycans, extends systemic circulation of biomimetic NPs by inhibiting phagocytosis by macrophages. Research has also shown that the structure of RBC membranes makes them convenient for trapping bacterial exotoxins [20]. Additionally, anucleate nature of erythrocytes simplifies membrane isolation and reduces contamination during production.

Leukocytes are another type of cell widely used in the synthesis of biomimetic NPs. Their membrane properties, such as LFA-1 (lymphocyte function-associated antigen 1) and chemokine receptors (e.g., CCR2, CXCR4), enable them to traverse endothelial barriers, facilitating applications involving infection site homing [22].

There are also biomimetic NPs based on platelets. Platelet membranes display surface receptors, including glycoprotein Ib-IX-V complex, integrin αIIbβ3, and toll-like receptors, which mediate vascular adhesion and pathogen recognition [46].

In addition, modern technologies have opened up the use of cancer cell membrane-coated NPs. These NPs carry tumor-associated antigens (TAAs), adhesion molecules, and immune checkpoint ligands (e.g., PD-L1), which promote immune evasion and prolonged circulation [47,48].

Finally, the mesenchymal stem cells express CXCR4, VLA-4, and multiple integrins, which enable homing to injury or inflammation sites. Their intrinsic immunomodulatory profile includes suppression of pro-inflammatory cytokine release and promotion of tissue repair, making them promising candidates for infectious disease therapy [49].

Bacterial cells represent an alternative source of coating material for NPs. Recent evidence indicates that bacterial vesicles possess native surface antigenic patterns capable of modulating immune responses and penetrating biofilms. Consequently, NPs coated with these membranes, which present pathogen-specific antigens, are emerging as promising platforms for developing effective vaccines against a range of bacterial infections [19].

Although the current technology allows using viral membranes to coat NPs, these particles are likely to keep the antigenic structure of the virus. While this can be advantageous for receptor-specific targeting, the risk of retaining immunogenic or pathogenic epitopes necessitates careful antigen removal or modification [23]. This favorable internalization profile is tempered by the challenge of mitigating off-target effects and unintended biological interactions. Despite their considerable advantages, the clinical translation of cell membrane-coated NPs faces several hurdles. A primary challenge lies in preserving membrane integrity during isolation, as conventional extraction techniques often employ harsh conditions that can compromise native protein structure and function. Consequently, the development of efficient purification protocols is critical for advancing the use of cell-derived membrane coatings. Further limitations concern manufacturing scalability and cost. Existing methods for fabricating biomimetic NPs often suffer from low drug-loading efficiency and rely on complex, expensive equipment. The process is inherently resource-intensive, requiring substantial cell quantities to yield sufficient membrane material. Sourcing membranes or proteins from blood cells introduces additional expenses and supply chain complexities related to securing adequate blood supplies. Therefore, pioneering cost-effective and scalable production strategies is essential to realizing the full therapeutic potential of this platform [22].

## 7. Applications for Cell Membrane-Coated NPs

### 7.1. Absorbing Toxins to Disarm Bacteria

The spread of antibiotic resistance is reflected in a rise in cases of infection by methicillin-resistant *Staphylococcus aureus* (MRSA), causing such diseases as sepsis and endocarditis. The toxins released by these bacteria such as α-toxin (hemolysin) play a significant role in the development of most MRSA-related symptoms, making the neutralization of these toxins crucial to the treatment [50]. One study suggested biomimetic NPs based on red blood cell (RBC) and Fe_3_O_4_ NPs cores–RBC@Fe_3_O_4_. Due to the specific structure of red blood cells, which extends the circulation half-life of the NPs in the bloodstream, they act as a molecular ”decoy” for the MRSA toxin, which has an affinity for human red blood cells. This construct reduced the concentration of the toxin secreted by the bacteria and secured the site of invasion [11].

This strategy can use biomimetic NPs based not only on RBCs but also other types of cells, such as macrophages. Endotoxins produced by bacteria may be released upon cell division, cell death, or antibiotic treatment. Macrophages are capable of binding bacterial LPS due to the pattern recognition receptors (PRRs), such as TLR4-MD-2 complexes. Using NPs with a preserved surface pattern of the macrophages that were used to produce them allowed endotoxin neutralization during fatal infections by Gram-negative bacteria [46]. The same mechanism was used with the membranes of other cells, such as platelets. Platelets are an important target for *S. aureus* α-toxin because they express disintegrin and the α-toxin receptor, metalloprotease domain-containing protein 10 (ADAM10), on their surface membrane. This enabled the successful use of platelet-based NPs for toxin neutralization, cytoprotection, and increased the organism’s resistance to invasion [51].

An interesting variation in NPs with red blood cell (RBC) membranes was observed. This multifunctional construct absorbed exotoxins from pathogenic bacteria. In addition, it contained a gelatin layer, sensitive to gelatinase secreted by invading bacteria, covering the core of the NPs with the active drug. When the NPs approached the site of bacterial invasion, the covering layer was destroyed by gelatinase, releasing the drug in close proximity and disrupting the bacterial membrane structure [52]. This demonstrates the versatility of NPs, which can perform multiple functions simultaneously.

### 7.2. Delivery of Antibiotics and Other Drugs to the Targets

A paramount application of biomimetic NPs is targeted drug delivery. These particles function as precision platforms, leveraging membrane-derived homing ligands and innate immune evasion capabilities to significantly enhance therapeutic concentration at specific sites of infection.

Recent studies used various types of cells to form NPs and deliver antibacterial substances in a more targeted manner. For instance, pulmonary epithelial cell membranes were used to synthesize biomimetic NPs equipped with curcumin molecules. Studies were then conducted to show the possible internalization of these NPs into the epithelial layer while avoiding absorption by macrophages. Compared to NPs systems coated with lipids and polymers, coating with epithelial membrane showed a significant increase in NPs uptake into pulmonary epithelial cells. The presence of proteins in biological membranes likely drove enhanced internalization [53].

Furthermore, platelet membranes were utilized to generate biomimetic NPs comprising the PLT@Ag-MOF-Vanc system, which consists of silver NPs and metal–organic frameworks loaded with the antibiotic vancomycin. The encapsulation and loading efficiency of vancomycin were achieved at 81.0 and 64.7%. This system was employed in a model of MRSA pneumonia. The membranes demonstrated favorable biocompatibility by diminishing the recognition and clearance of the reticuloendothelial system and enabling more effective delivery of NPs. The combination of Ag-MOF’s bactericidal properties and vancomycin resulted in the inhibition of MRSA growth. The efficacy of the synergistic bactericidal therapy was found to exceed that of vancomycin administered as a monotherapy [54].

The therapeutic approach encompassed not only the administration of antibiotics but also the utilization of antimicrobial peptides as a potential treatment for bacterial infections. In a recent study, a system of KLA-neutrophil NPs was obtained from neutrophil membranes and poly(lactic-co-glycolic acid) (PLGA) NPs cores loaded with the broad-spectrum aminoglycoside antibiotic gentamicin and fixed molecules of the antibacterial peptide KLA. The neutrophil membrane inhibited the phagocytosis of NPs by macrophages, thereby increasing circulation and blood compatibility. Furthermore, the antibiotic and bactericidal peptide molecules retained their functional efficacy, exerting a deleterious effect on the growth and reproduction of the pathogenic bacteria [55].

Another study suggested a system combining membranes derived from mesenchymal stem cells originating from bone marrow and a metal–organic framework composed of silver, loaded with FPS-ZM1 and meropenem. The loading capacity and encapsulation efficiency of the FPS-ZM1 were evaluated as 20% and 84%, while those of meropenem were 21% and 61%. The efficacy of these NPs was assured by the effects of their components: FPS-ZM1, an inhibitor of the receptor for advanced glycation end products, reduced inflammation, while meropenem is an antibiotic inhibiting bacterial cell synthesis. In turn, mesenchymal bone marrow stem cells express a variety of chemokine receptors and integrins on their surface, which allows the NPs to specifically target areas of inflammation and damage [56].

Besides antibiotics, nanoformulations can be used to deliver molecules such as mRNA and plasmid DNA. Incorporation of plasmid DNA carrying the antimicrobial gene LL37 into a zinc metal–organic framework and subsequent coating with a macrophage membrane (loading efficiency 75%), facilitated targeted delivery of the LL37 gene and the subsequent production of antimicrobial peptides within the body’s macrophage cells [57]. Furthermore, utilization of NPs that use viral membranes with proteins expressed on their surface has been successful for delivering mRNA molecules, resulting in effective expression of the encoded protein [58]. Consequently, the delivery of programmed antigens may facilitate the immune response to bacterial infections.

As previously mentioned, bacteria have the capacity to evade antibiotics by producing a biofilm. The utilization of biomimetic NPs facilitates the penetration of the biofilm matrix. Biomimetic NPs for this task were based on the macrophage membranes. These NPs carry PLGA and molecule named FOT (NO donor), and R837, which serves as a TLR7 agonist, within their core structure. Following interaction with the body’s macrophages, the release of FOT and R837 ensued. The released FOT would react with glutathione (GSH) in the milieu, leading to the production of NO in the biofilm matrix. This, in turn, resulted in the destruction of the biofilm and the eradication of bacteria. This process attracted macrophages, prompting them to phagocytose planktonic bacteria detached from the biofilm. This, in turn, contributed to the therapeutic effect [59].

### 7.3. Photothermal Application of Biomimetic NPs

One of the most promising applications for biomimetic NPs is the delivery and activation of photothermal molecules [60,61].

Consequently, membranes of macrophages pre-activated for pulmonary tuberculosis were used to create biomimetic NPs carrying phototherapeutic agents. The incorporation of macrophage membranes has been instrumental in facilitating specific ligand-receptor binding, thereby ensuring prolonged circulation of the drug. Following the binding of the NPs and the activation of NIR-II AIEgen, the biomimetic NPs exhibited remarkable bactericidal activity against *Mycobacterium tuberculosis* [62,63]. NPs derived from natural hair and subsequently coated with murine macrophage membranes (RAWM) have also been developed as a biomimetic platform. Upon near-infrared laser irradiation, these hybrid RAWM NPs exhibited efficient photothermal conversion (42.13%), achieving a better photothermal curative effect through enhanced bacterial killing in vivo, while maintaining biocompatibility. Such findings highlight the potential of membrane-coated photothermal nanoplatforms as adjunctive antibacterial therapies [64].

Biomimetic NPs that included neutrophil membranes and *Acinetobacter baumannii* virulence factors were tested as a vaccine candidate. This pathogen can cause various diseases, including ventilator-associated pneumonia, meningitis, and sepsis, as well as infections of the urinary tract, skin, and other soft tissues [65]. The vaccine demonstrated significant protective efficacy, as evidenced by a substantial increase in survival rates and a reduction in signs of acute inflammation [66].

## 8. Challenges and Future Perspectives

The transition of biological and biomimetic nanoparticles from promising experimental platforms to reliable clinical therapeutics is hampered by several persistent challenges [45]. Scaling up manufacturing while ensuring batch-to-batch consistency remains a primary obstacle, especially for natural EVs. Current methods for isolating natural EVs from mammalian or bacterial cells also suffer from low yields and high sensitivity to culture conditions. This inherent variability directly impacts critical quality attributes, including nanovesicle composition and stability, making it difficult to establish the standardized, GMP-compliant production processes required for clinical use. Manufacturing of bottom-up, fully synthetic, hybrid and other fabricated types of NPs have substantial advantages, overcoming major technological hurdles, demonstrating markedly higher yields and batch-to-batch consistency. However, other hurdles persist, such as achieving efficient vesicle-core fusion, preserving correct membrane protein orientation, long-term storage conditions etc. [12,14], all essential for creating uniform NP batches with predictable behavior and standardized production pipelines.

Beyond production, the complex interplay between these NPs and biological systems presents another layer of uncertainty. For instance, OMV-based systems may provoke excessive immune activation due to residual PAMPs, while exosome formulations might carry a heterogeneous mix of nucleic acids, including DNA, with unknown biological consequences or risks of inflammatory responses. A deeper understanding of their effects in the human body, including pharmacokinetics, biodistribution, and organ accumulation, is crucial and still largely lacking.

Despite these hurdles, the path forward is becoming clearer. Promising strategies to overcome current limitations include investing in next-generation biomanufacturing. Technologies such as microfluidic coating platforms, more efficient vesicle isolation methods, and even cell-free membrane synthesis could dramatically improve production efficiency and reduce the variability that currently plagues the field (Table 2).

## 9. Conclusions

The accelerating spread of antibiotic resistance presents one of the most urgent challenges in modern infectious disease management. Biological NPs such as exosomes, outer membrane vesicles, and cell membrane-coated nanostructures offer a versatile therapeutic platform with the capacity for targeted delivery, immune modulation, biofilm penetration, and toxin neutralization. Nevertheless, this technology raises concerns, including the standardization of NPs synthesis, scaling of membrane isolation processes, batch-to-batch reproducibility, and the establishment of regulatory safety frameworks tailored to hybrid biological–synthetic materials. Advancing in these directions through coordinated interdisciplinary research will facilitate the safe and effective clinical implementation of biological nanoparticles in antibacterial therapy. Notwithstanding the prevailing challenges, biological NPs are heralding a new era in antimicrobial therapy, offering customized solutions to combat the most perilous pathogens today. Continued innovation in bio-interface engineering, manufacturing, and in vivo validation will be critical to realizing their full clinical impact.

## Figures and Tables

**Table 1:** Comparative characteristics of the main types of biological nanoparticles.

Feature	Exosomes	Outer Membrane Vesicles (OMVs)	Cell Membrane-Coated Nanoparticles (CMNPs)
Origin	Endosomal pathway of eukaryotic cells (multivesicular bodies → plasma membrane fusion) [12]	Blebbing of outer membrane of Gram-negative bacteria [13]	Synthetic nanoparticle cores cloaked with plasma membranes from RBCs, WBCs, platelets, MSCs, bacteria, cancer cells, or viruses [8]
Typical Size	30–150 nm [14]	50–250 nm [13]	50–200 nm (depends on NP core size and membrane source)
Biocompatibility	High biocompatibility, as they originate from endogenous cellular membranes [15]	Relatively low biocompatibility due to the presence of lipopolysaccharides (LPS) and other bacterial immunostimulatory components [13]	Moderate to high biocompatibility, depending on the membrane source; membranes derived from mammalian cells (e.g., RBCs, leukocytes, MSCs), extent of cell membrane-coating, display good hemocompatibility and immune tolerance, whereas bacterial or cancer cell coatings may elicit stronger immune responses
Surface Markers/ Composition	Tetraspanins (CD9, CD63, CD81), adhesion molecules, lipids, nucleic acids (miRNA, mRNA, proteins) [12]	Lipopolysaccharides (LPS), outer membrane proteins (OMPs), phospholipids, peptidoglycan fragments, bacterial DNA/RNA, virulence factors [13]	Retain native membrane proteins, glycosylation, receptors (e.g., CD47 on RBCs, CCR2/CXCR4 on leukocytes, ADAM10 on platelets, TAAs on cancer cells, PAMPs on bacterial membranes) [8,16,17,18,19,20]
Immunogenicity	Low (self-derived, minimal immune activation) [15]	High (LPS and bacterial antigens strongly activate immune system) [21]	Variable—depends on source membrane (e.g., RBC low immunogenicity, bacterial/cancer higher)
Advantages	Natural carriers, low toxicity, efficient intercellular communication, can cross barriers (e.g., BBB) [15]	Strong innate and adaptive immune activation, mimic pathogenic bacteria, efficient antigen presentation [21]	Modular design, tunable NP core [8], prolonged circulation [16], immune evasion [22], toxin absorption [17], pathogen/tissue specificity [23]
Limitations	Difficult large-scale isolation, heterogeneity, low yield, purification challenges [14]	Endotoxin-associated toxicity, stability issues, possible off-target inflammation [21]	Complex fabrication, scalability issues, membrane extraction efficiency, regulatory challenges [24]

**Table 2:** Summary of types of biological nanoparticles, their properties and applications.

Type of Biological Nanoparticle	Origin/Composition	Key Functional Properties	Representative Applications	Advantages	Limitations
Exosomes	Derived from eukaryotic cells; composed of lipid bilayers containing proteins (CD9, CD63, CD81), RNAs, and lipids [12]	Natural carriers; biocompatible; capable of intercellular communication and immune modulation	1. Antibiotic delivery (linezolid, rifampicin) against *S. aureus*, *M. tuberculosis* [33,34] 2. Delivery of genetic material (siRNA, miRNA) 3. Vaccine development and antigen presentation (e.g., *M. tuberculosis*) [35]	Low immunogenicity; endogenous origin; ability to cross biological barriers [34]	Low yield and isolation efficiency; heterogeneity; challenges in large-scale production [14]
Outer Membrane Vesicles (OMVs)	Naturally secreted by Gram-negative bacteria; composed of outer membrane lipids, lipopolysaccharides (LPS), proteins, and DNA/RNA	High immunogenicity; mimic bacterial antigens; self-adjuvant properties	1. Vaccine platforms (e.g., *Pseudomonas aeruginosa*, *Acinetobacter Baumannii*) [40,41] 2. Antibiotic delivery (e.g., levofloxacin) against *Pseudomonas aeruginosa*, *Klebsiella pneumoniae* and *Escherichia coli* [38,39] 3. Immunomodulatory and adjuvant applications [42]	Strong immune activation; natural pathogen mimicry [21]; easy genetic engineering	Endotoxin-related toxicity (LPS); potential inflammatory responses; stability concerns
Cell Membrane-Coated Nanoparticles (CMNPs)	Synthetic nanoparticle cores coated with biological membranes (RBCs, WBCs, platelets, MSCs, bacterial or cancer membranes)	Combine physical tunability of synthetic NPs with biological functions of native membranes	1. Toxin neutralization (*S. aureus* α-toxin, LPS, etc.) [17,47,48] 2. Photothermal and photodynamic antibacterial therapy (e.g., *Mycobacterium tuberculosis*) [58,59] 3. Biofilm disruption and infection-site targeting [55] 4. Co-delivery of antibiotics and adjuvants (MRSA, *K. pneumonia,* etc.) [51,60]	Prolonged circulation [51]; immune evasion; pathogen-specific targeting [52]; multifunctionality [49]	Complex fabrication; membrane extraction efficiency; scalability and reproducibility issues [24]

## Data Availability

No new data were created or analyzed in this study. Data sharing is not applicable to this article.
